# Supplementary material for: The Association between Salt and Potential Mediators of the Gastric Precancerous Process
Source: Cancers (Basel). 2019 Apr 15;11(4):535. doi: 10.3390/cancers11040535 (PMC6520685; doi:10.3390/cancers11040535)
Supplement: Supplementary file 1 [file cancers-11-00535-s001.pdf]

cancers

MDPI

Supplementary Materials: The Association Between Salt and Potential Mediators of the Gastric Precancerous Process

Susan Thapa, Lori A. Fischbach, Robert Delongchamp, Mohammed F. Faramawi and Mohammed Orloff

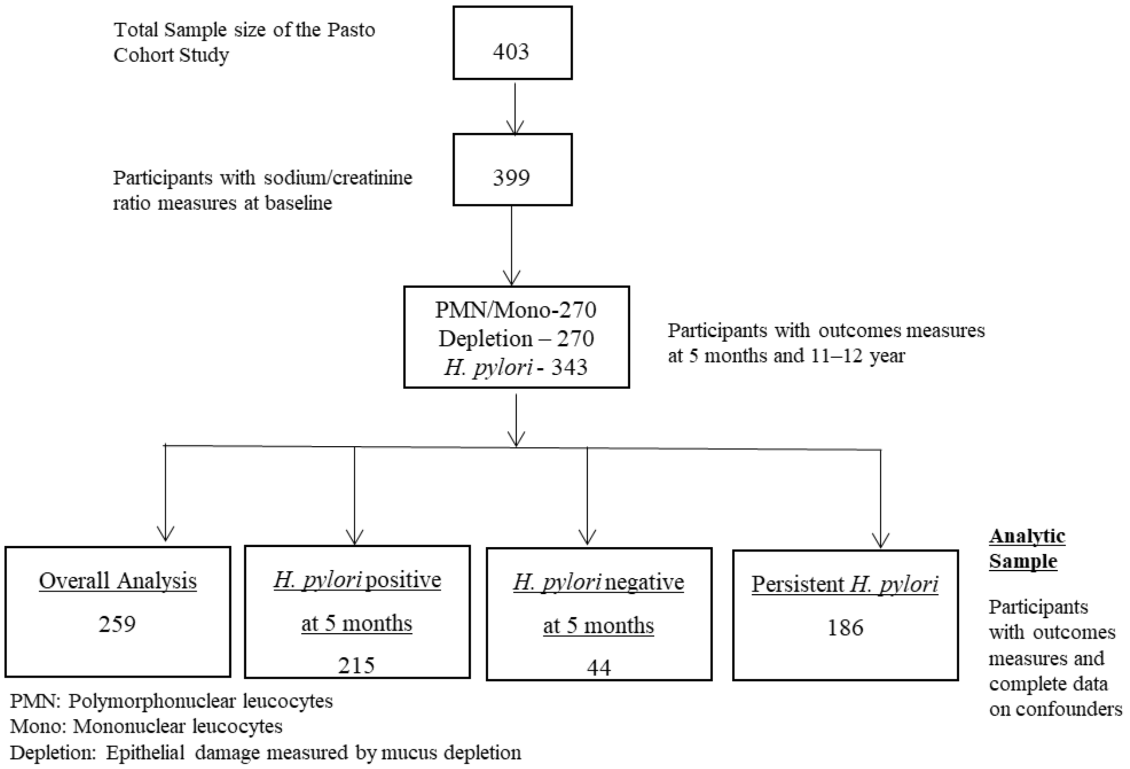

Figure S1. Sample sizes for the analyses of the estimated effect of sodium/creatinine ratio on gastric inflammation, epithelial damage, *H. pylori* infection density, and gastric epithelial cell proliferation.

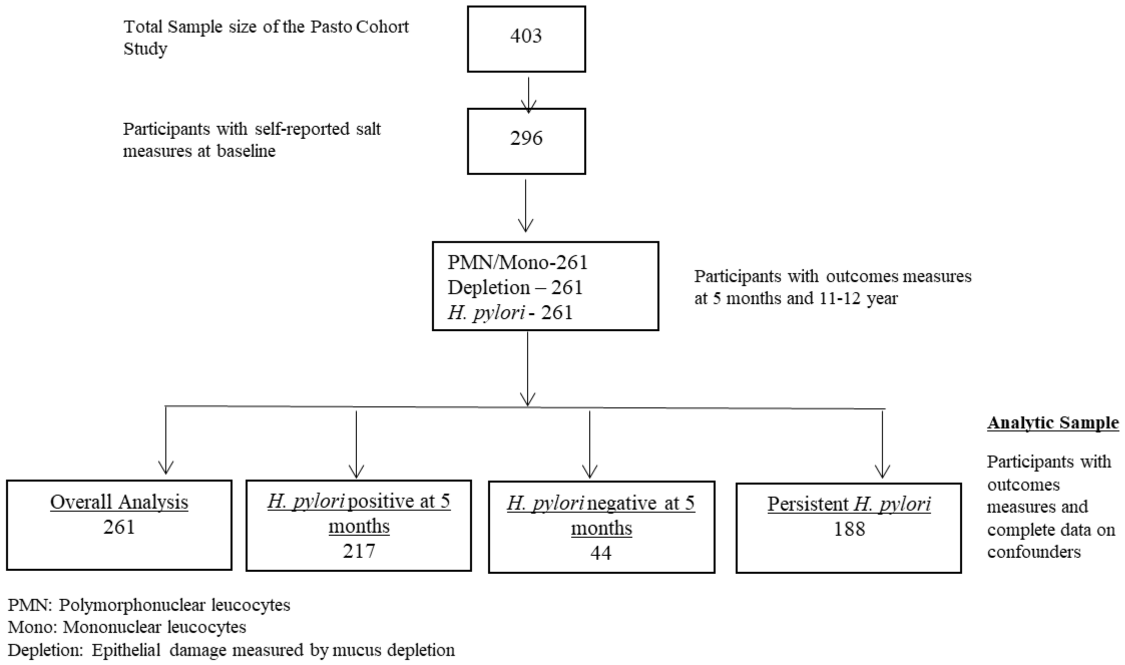

Figure S2. Sample sizes for the analyses of the estimated effect of self-reported salt intake (frequency of adding salt to foods and total added salt) on gastric inflammation, epithelial damage, *H. pylori* infection density, and gastric epithelial cell proliferation.

Table S1. Results of linear regression for the estimated effect of salt intake measured as the frequency of adding salt to foods at baseline on gastric inflammation and epithelial damage.[a]

Outcome		Level	Adjusted ß (95% Confidence Intervals) [b] Overall (*n* = 261)	*H. pylori* Positive at 5 Months (*n* = 217)	*H. pylori* Negative at 5 Months (*n* = 44)	Persistent *H. pylori* Infection (Positive at 5 Months and at 11–12 Years) (*n* = 188)
Gastric Inflammation	Change in average inflammation (5 months vs 11–12 years)	PMN	0.155 (−0.065, 0.376)	0.121 (−0.448, 0.690)	0.101 (−0.091, 0.293)	0.155 (−0.065, 0.376)
		Mononuclear	0.036 (−0.081, 0.152)	0.377 (−0.035, 0.790)	0.023 (−0.074, 0.119)	0.036 (−0.081, 0.152)
		Combined	0.096 (−0.061, 0.252)	0.249 (−0.218, 0.717)	0.062 (−0.065, 0.189)	0.096 (−0.061, 0.252)
	Change in maximum inflammation (5 months vs 11–12 years)	PMN	0.092 (−0.151, 0.334)	0.159 (−0.511, 0.829)	0.001 (−0.207, 0.209)	0.092 (−0.151, 0.334)
		Mononuclear	0.068 (−0.106, 0.241)	0.160 (−0.275, 0.595)	0.040 (−0.120, 0.200)	0.068 (−0.106, 0.241)
		Combined	0.040 (−0.150, 0.231)	0.235 (−0.256, 0.726)	-0.031 (−0.198, 0.137)	0.040 (−0.150, 0.231)
Epithelial Damage	Change in average Epithelial damage (5 vs 11–12 years)		0.088 (−0.137, 0.313)	0.471 (−0.186, 1.129)	0.049 (−0.163, 0.261)	0.088 (−0.137, 0.313)
	Change in maximum Epithelial Damage (5 vs 11–12 years)		0.049 (−0.212, 0.310)	0.659 (−0.113, 1.432)	−0.013 (−0.239, 0.214)	0.049 (−0.212, 0.310)
Interaction between gastric inflammation and epithelial damage	Inflammation at 5 months * Mucus Depletion at 11–12 years	PMN	−0.079 (−0.701, 0.543)	0.249 (−0.410, 0.908)	0.599 (−0.165, 1.363)	0.153 (−0.516, 0.823)
		Mononuclear	0.038 (−0.365, 0.440)	0.258 (−0.188, 0.705)	0.257 (−0.581, 1.094)	0.194 (−0.235, 0.622)
		Combined	0.017 (−0.540, 0.574)	0.271 (−0.341, 0.884)	0.675 (−0.324, 1.673)	0.179 (−0.408, 0.766)
	Inflammation at 11-12 years * Mucus Depletion at 5 months	PMN	−0.141 (−0.664, 0.381)	0.225 (−0.343, 0.792)	0.030 (−0.701, 0.761)	0.156 (−0.442, 0.754)
		Mononuclear	−0.171 (−0.615, 0.273)	0.221 (−0.258, 0.70)	−0.619 (−1.238, 0.001)	0.191 (−0.334, 0.715)
		Combined	−0.271 (−0.778, 0.235)	0.152 (−0.392, 0.696)	−0.560 (−1.329, 0.208)	0.091 (−0.502, 0.683)
H. pylori infection density	Change in average *H. pylori* density (5 months vs 11–12 years)		0.051 (−0.178, 0.281)	0.137 (−0.396, 0.670)		0.051 (−0.178, 0.281)
	Change in max *H. pylori* density (5 months vs 11–12 years)		0.091 (−0.183, 0.364)	0.233 (−0.392, 0.857)		0.091 (−0.183, 0.364)
Interaction between density of *H. pylori* infection,	Density of *H. pylori* infection at 5 months * Mucus Depletion at 5 months		−0.227 (−0.654, 0.201)	0.064 (−0.384, 0.512)		0.065 (−0.435, 0.565)

inflammation and epithelial damage	Density of *H. pylori* infection at 11-12 years * Mucus Depletion at 11–12 years		−0.022 (−0.686, 0.642)	0.103 (−0.612, 0.817)	0.125 (−0.499, 0.749)
	Density of *H. pylori* infection at 5 months * Mucus Depletion at 11–12 years		−0.214 (−0.808, 0.380)	0.277 (−0.317, 0.871)	0.141 (−0.420, 0.701)
	Density of *H. pylori* infection at 11–12 years *Mucus Depletion at 5 months		−0.068 (−0.571, 0.434)	0.278 (−0.274, 0.830)	0.173 (−0.389, 0.735)
	Density of *H. pylori* infection at 5 months * Inflammation at 5 months * Mucus Depletion at 11–12 years	PMN	−0.297 (−1.735, 1.140)	0.695 (−0.862, 2.252)	0.503 (−1.134, 2.139)
		Mononuclear	−0.265 (−1.328, 0.797)	0.520 (−0.620, 1.659)	0.370 (−0.817, 1.557)
		Combined	−0.312 (−1.731, 1.107)	0.751 (−0.757, 2.258)	0.574 (−0.978, 2.126)
	Density of *H. pylori* infection at 11–12 years * Inflammation at 11–12 years * Mucus Depletion at 5 months	PMN	−0.073 (−1.582, 1.435)	0.987 (−0.686, 2.659)	0.748 (−1.001, 2.498)
		Mononuclear	0.113 (−1.180, 1.406)	1.068 (−0.365, 2.502)	0.888 (−0.602, 2.379)
		Combined	−0.151 (−1.661, 1.358)	0.899 (−0.772, 2.569)	0.627 (−1.107, 2.362)

[a] The change in score for the outcome per increase in salt intake frequency (the three categories of salt intake include: rarely or never adds salt to foods, occasionally adds salt to foods, and always or frequently adds salt to foods).

[b] Adjusted for age, car ownership, and fresh fruit and vegetable intake; for changes in gastric inflammation, epithelial damage, and density of *H. pylori* infection; and for baseline measures.

Table S2. Results of linear regression for the estimated effect of salt intake measured as total salt added to food at baseline on gastric inflammation and epithelial damage.

Outcome		Level	Adjusted ß (95% Confidence Intervals) [a]			
			**Overall (*n* = 261)**	***H. pylori* Positive at 5 Months (*n* = 217)**	***H. pylori* Negative at 5 Months (*n* = 44)**	**Persistent *H. pylori* Infection (Positive at 5 Months and at 11–12 Years) (*n* = 188)**
Gastric Inflammation	Change in average inflammation (5 months vs 11–12 years)	PMN	−0.009 (−0.137, 0.119)	0.010 (−0.120, 0.140)	−0.214 (−0.593, 0.164)	−0.007 (−0.117, 0.104)
		Mononuclear	−0.008 (−0.079, 0.062)	0.014 (−0.055, 0.082)	−0.143 (−0.420, 0.133)	0.007 (−0.049, 0.062)
		Combined	−0.011 (−0.102, 0.080)	0.011 (−0.081, 0.103)	−0.192 (−0.506, 0.121)	−0.001 (−0.074, 0.072)
	Change in maximum inflammation (5 months vs 11–12 years)	PMN	−0.027 (−0.173, 0.119)	0.004 (−0.138, 0.146)	−0.301 (−0.744, 0.141)	−0.010 (−0.129, 0.109)
		Mononuclear	0.042 (−0.054, 0.137)	0.040 (−0.062, 0.142)	−0.028 (−0.313, 0.257)	0.027 (−0.064, 0.119)
		Combined	−0.022 (−0.130, 0.086)	−0.009 (−0.121, 0.103)	−0.199 (−0.528, 0.129)	−0.028 (−0.124, 0.068)
Epithelial Damage	Change in average Epithelial damage (5 vs 11–12 years)		−0.019 (−0.149, 0.110)	−0.003 (−0.135, 0.129)	−0.122 (−0.56, 0.317)	−0.020 (−0.141, 0.101)
	Change in maximum Epithelial Damage (5 vs 11–12 years)		0.029 (−0.121, 0.179)	0.027 (−0.127, 0.180)	0.028 (−0.495, 0.551)	0.008 (−0.121, 0.138)
Interaction between gastric inflammation and epithelial damage	Inflammation at 5 months * Mucus Depletion at 11–12 years	PMN	0.165 (−0.210, 0.540)	0.141 (−0.246, 0.527)	0.477 (0.009, 0.945)	0.074 (−0.309, 0.457)
		Mononuclear	−0.055 (−0.298, 0.188)	−0.035 (−0.298, 0.228)	0.065 (−0.461, 0.591)	−0.090 (−0.335, 0.155)
		Combined	0.135 (−0.201, 0.471)	0.123 (−0.236, 0.483)	0.450 (−0.172, 1.073)	0.050 (−0.286, 0.386)
	Inflammation at 11–12 years * Mucus Depletion at 5 months	PMN	−0.040 (−0.355, 0.276)	0.004 (−0.329, 0.337)	0.023 (−0.433, 0.480)	−0.050 (−0.392, 0.292)
		Mononuclear	−0.058 (−0.326, 0.210)	−0.028 (−0.309, 0.254)	−0.029 (−0.436, 0.378)	−0.086 (−0.386, 0.214)
		Combined	−0.071 (−0.377, 0.236)	−0.030 (−0.350, 0.289)	−0.003 (−0.497, 0.490)	−0.092 (−0.431, 0.246)
H. pylori infection density	Change in average *H. pylori* density (5 months vs 11–12 years)		−0.056 (−0.194, 0.082)	−0.055 (−0.189, 0.079)		−0.081 (−0.203, 0.041)
	Change in max *H. pylori* density (5 months vs 11–12 years)		−0.012 (−0.173, 0.150)	0.001 (−0.159, 0.161)		−0.035 (−0.158, 0.089)
Interaction between density of *H.*	Density of *H. pylori* infection at 5 months * Mucus Depletion at 5 months		−0.093 (−0.349, 0.164)	−0.093 (−0.354, 0.169)		−0.093 (−0.378, 0.191)

pylori infection, inflammation and epithelial damage	Density of *H. pylori* infection at 11–12 years * Mucus Depletion at 11–12 years		−0.057 (−0.456, 0.341)	−0.011 (−0.430, 0.409)		−0.082 (−0.438, 0.275)
	Density of *H. pylori* infection at 5 months * Mucus Depletion at 11–12 years		0.017 (−0.340, 0.373)	0.053 (−0.295, 0.401)		−0.008 (−0.327, 0.311)
	Density of *H. pylori* infection at 11–12 years * Mucus Depletion at 5 months		−0.145 (−0.448, 0.158)	−0.135 (−0.459, 0.189)		−0.179 (−0.499, 0.142)
	Density of *H. pylori* infection at 5 months * Inflammation at 5 months * Mucus Depletion at 11–12 years	PMN	0.258 (−0.605, 1.120)	0.380 (−0.530, 1.291)		0.269 (−0.663, 1.201)
		Mononuclear	−0.053 (−0.691, 0.585)	0.009 (−0.659, 0.676)		−0.081 (−0.757, 0.595)
		Combined	0.242 (−0.610, 1.093)	0.367 (−0.514, 1.249)		0.249 (−0.635, 1.133)
	Density of *H. pylori* infection at 11–12 years * Inflammation at 11–12 years * Mucus Depletion at 5 months	PMN	−0.354 (−1.263, 0.555)	−0.288 (−1.272, 0.696)		−0.398 (−1.399, 0.602)
		Mononuclear	−0.366 (−1.145, 0.413)	−0.344 (−1.187, 0.500)		−0.453 (−1.306, 0.400)
		Combined	−0.414 (−1.324, 0.495)	−0.361 (−1.343, 0.620)		−0.482 (−1.473, 0.508)

[a] The change in score for the outcome per increase in salt intake in grams. [b] Adjusted for age, car ownership, and fresh fruit and vegetable intake; for changes in gastric inflammation, epithelial damage, and density of *H. pylori* infection; and for baseline measures.

© 2019 by the authors. Submitted for possible open access publication under the terms and conditions of the Creative Commons Attribution (CC BY) license (http://creativecommons.org/licenses/by/4.0/).
